# Development and biological applications of sulfur–triazole exchange (SuTEx) chemistry

**DOI:** 10.1039/d0cb00180e

**Published:** 2021-01-19

**Authors:** Adam L. Borne, Jeffrey W. Brulet, Kun Yuan, Ku-Lung Hsu

**Affiliations:** Department of Pharmacology, University of Virginia School of Medicine Charlottesville Virginia 22908 USA; Department of Chemistry, University of Virginia McCormick Road, P.O. Box 400319 Charlottesville Virginia 22904 USA kenhsu@virginia.edu +1-434-297-4864; University of Virginia Cancer Center, University of Virginia Charlottesville VA 22903 USA; Department of Molecular Physiology and Biological Physics, University of Virginia Charlottesville Virginia 22908 USA

## Abstract

Sulfur electrophiles constitute an important class of covalent small molecules that have found widespread applications in synthetic chemistry and chemical biology. Various electrophilic scaffolds, including sulfonyl fluorides and arylfluorosulfates as recent examples, have been applied for protein bioconjugation to probe ligand sites amenable for chemical proteomics and drug discovery. In this review, we describe the development of sulfonyl-triazoles as a new class of electrophiles for sulfur–triazole exchange (SuTEx) chemistry. SuTEx achieves covalent reaction with protein sites through irreversible modification of a residue with an adduct group (AG) upon departure of a leaving group (LG). A principal differentiator of SuTEx from other chemotypes is the selection of a triazole heterocycle as the LG, which introduces additional capabilities for tuning the sulfur electrophile. We describe the opportunities afforded by modifications to the LG and AG alone or in tandem to facilitate nucleophilic substitution reactions at the SO_2_ center in cell lysates and live cells. As a result of these features, SuTEx serves as an efficient platform for developing chemical probes with tunable bioactivity to study novel nucleophilic sites on established and poorly annotated protein targets. Here, we highlight a suite of biological applications for the SuTEx electrophile and discuss future goals for this enabling covalent chemistry.

## Abstract 2

This review discusses the development of sulfonyl–triazoles and highlights the merits and opportunities for deploying this sulfur electrophile for biological discovery.

## Graphical abstract

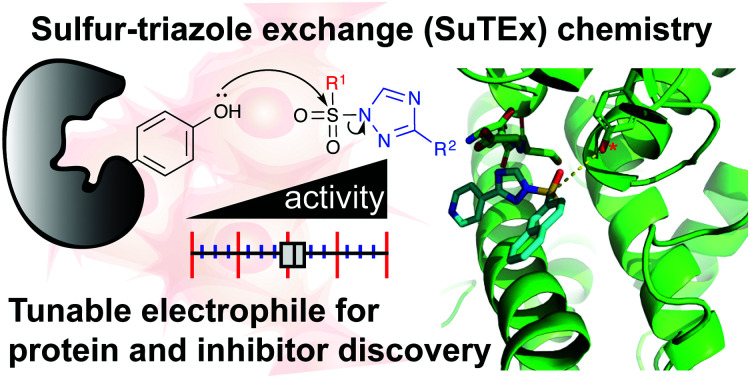

## Introduction

Small molecule probes are enabling tools for understanding protein function in cells and tissues under healthy and diseased states.^1,2^ Chemical probes offer a complementary approach to genetic methods for investigating biological pathways and testing therapeutic hypotheses.^3^ These tools can reveal pharmacological effects of protein modulation that are rapid, reversible, and universal with respect to sample type.^1^ Small molecule probes have facilitated the annotation of function to poorly characterized proteins,^4^ identification of protein inhibitors^5^ and activators^6,7^ of existing^8,9^ or new therapeutic targets,^10^ and discovery of new mechanisms of action for modulating protein activity (*e.g.* PROteolysis TArgeting Chimeras or PROTACs^11–15^). Chemical probes can also provide key insights into selectivity and off-target activity of compounds to develop safer drugs and, in some instances, offer molecular insights into unexpected toxicity of clinical candidates (*e.g.* BIA 10-2474^16^).

**Fig. 1:**
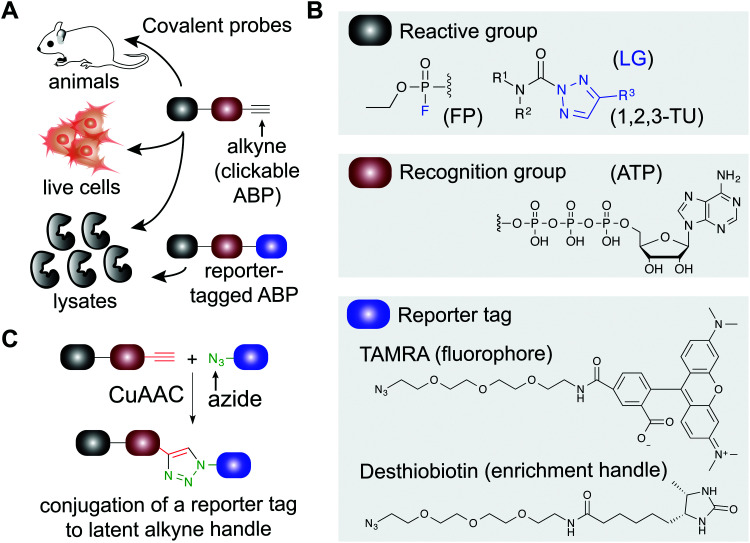
Applications of covalent probes for activity-based protein profiling (ABPP). (A) Covalent probes are enabling and versatile tools for functional studies of proteins in complex biological systems. (B) The basic components of an activity-based probe (ABP) include: a reactive group, a recognition group for binding, and a reporter tag. Representative examples of each component are shown. The reactive group is utilized to form a covalent bond between the ABP and target protein. For example, electrophiles including fluorophosphonates and 1,2,3-triazole ureas can be used for enzymes that contain nucleophilic residues in catalytic domains of serine hydrolases. Recognition groups are generally based on natural substrates of enzymes and used to direct ABPs to protein sites through affinity for the probe structure. The reporter tag facilitates ABP-labeling events to be detected and measured. Fluorophores such as tetramethylrhodamine (TAMRA) are commonly used to visualized probe labeling. ABP-modified proteins can also be enriched for LC-MS analysis to identify and quantify target protein and binding site(s) using (desthio)biotin enrichment tags (see Fig. 2). (C) Typically, reporter-tagged ABPs are not well suited for studies in living systems because of poor cell permeability. Instead, alkynes are deployed as latent chemical handles to produce ‘clickable’ ABPs that can undergo copper(I)-catalyzed azide–alkyne [3+2] cycloaddition (CuAAC) to incorporate azide-modified reporter tags into ABP-labeled proteins for detection, enrichment, and identification from ABP treated cells and animals. LG: leaving group.

**Fig. 2:**
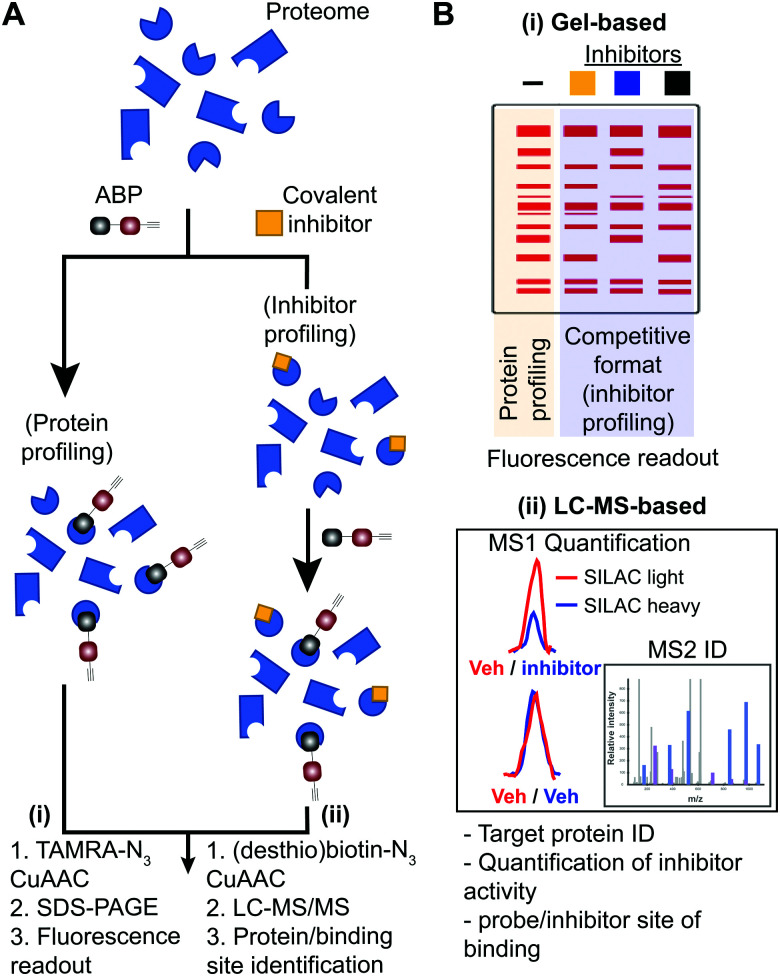
Workflow for standard and competitive ABPP analyses. (A) Proteomes from biological samples (cells, tissues, patient-derived material) are treated with an ABP that is selected based on targets of interest and results in labeling of active enzymes/proteins for protein profiling experiments. Potency and selectivity of inhibitors can be globally evaluated by labeling proteomes from inhibitor pretreated lysate or live cells/animals (after lysis or homogenization of cells or tissues) with ABP in a competitive inhibitor profiling experiment. The competitive ABPP workflow results in ABP labeling of uninhibited enzymes/proteins, which allows comparison of ABP profiles in the absence and presence of competitor to identify inhibitor targets through reductions or blockade of ABP labeling. (B) Detection of protein targets can be achieved using (i) in-gel fluorescence scanning that separates and visualizes ABP-modified proteins by molecular weight using SDS-PAGE, or (ii) LC-MS enrichment, identification, and quantification of ABP-modified proteins. Protein identification is generally performed on tryptic peptides derived from ABP-modified proteins enriched by avidin affinity chromatography. Trypsin digestion prior to affinity chromatography allows enrichment of ABP-modified peptides derived from target proteins to identify and quantify (when combined with SILAC or tandem mass tag (TMT)) site of binding for ABPs and inhibitors when using a competitive format. For clickable probes, alkynes are first reacted with an azide-reporter tag as described in Fig. 1.

Covalent small molecules constitute an important class of probes that exploit structural and reactivity features of protein sites to facilitate irreversible modification for activity-based protein profiling (ABPP) studies.^17,18^ ABPP assays are performed using a ‘probe’ that contains a reporter tag that achieves labeling through active site accessibility and reactivity of a nucleophilic amino acid (*e.g.* catalytic residue) to mediate labeling of functional proteins across the proteome (Fig. 1). ABPP probes can be designed with affinity labeling reagents to bind specific protein classes using, for example, fluorophosphonate probes^19^ and triazole ureas for the serine hydrolase superfamily^20^ (Fig. 1). ABPP studies can be deployed in a competitive format for inhibitor discovery by treating biological systems with a candidate small molecule ‘inhibitor’ or ‘ligand’ and comparing its protein activities, as quantified by probe labeling, with those of an untreated system (Fig. 2). The ability to screen candidate inhibitors against numerous proteins simultaneously *in vitro* and *in vivo* allows a more systematic evaluation of potency and selectivity in complex biological systems.^21^

Global covalent probes that broadly modify nucleophilic amino acids, as opposed to a particular protein class, have been developed to expand the utility of ABPP and chemical proteomics in general. These efforts have been facilitated by development of chemistry amenable for profiling cysteine^22–26^ (including covalent reversible electrophiles^27^), lysine,^28–31^ aspartate/glutamate,^32–34^ methionine,^35^ and tyrosine^36–42^ residues, and have led to new opportunities for developing ligands to modulate protein function.^14,15,23,28,43^ ABPP can be applied for discovery of protein electrophiles using nucleophilic hydrazine probes.^44^ Covalent probes can be adapted for studying post-translational modifications (PTM) including citrullination,^45^ methylation,^46^ phosphorylation,^36^ crotonolyation,^47^ palmitoylation,^48,49^ and glycosylation^50^ through direct and metabolic labeling methods. Functionalized inhibitor molecules and fragments bearing photoreactive groups have also been used for proteomic discovery of ligand sites that may not necessarily contain a nucleophilic residue.^51–54^

Despite advances in small molecule development, a vast majority of the human proteome lacks pharmacological probes.^55–57^ New chemical technologies to enable small-molecule discovery and pharmacological intervention of ‘undruggable’ targets are needed to reveal functional details of understudied proteins^58–60^ that are the mediators of human disease.^2,5,11–13,61–65^ Among compounds explored recently, sulfur electrophiles represent an important chemotype for advancing covalent probes in chemical biology and therapeutic discovery. Sulfonyl-fluorides^41^ (–SO_2_F) and fluorosulfates^66,67^ (–OSO_2_F) target a wide range of amino acids (*e.g.* serine,^68–70^ tyrosine,^39^ lysine,^29^ histidine^71^) and diverse protein targets (proteases,^68,69^ kinases,^29^ G-protein–coupled receptors (GPCRs^72^)) that are amenable to sulfur–fluoride exchange chemistry (SuFEx^73^). Sulfuramidimidoyl fluorides have recently emerged as a more selective variant of SuFEx for developing protein ligands.^74^ Applications of SuFEx probes include investigation of specific protein classes including kinases (*e.g.* XO44 for selectivity profiling of dasatinib^29^) as well as late-stage functionalization of phenol-containing drugs or drug candidates for developing new anticancer agents.^75^

In this review, we describe the development of sulfur–triazole exchange (SuTEx) chemistry as a new chemotype for advancing the tunability and chemical diversity of the sulfur electrophile. Although SuTEx shares common features with SuFEx, the deployment of a triazolide leaving group in the former chemistry enables new opportunities for medicinal chemistry, chemical biology, and potentially therapeutic discovery. Triazoles are readily accessible and can tolerate broad functional group diversity, which introduces an additional layer of tunability in design of sulfur electrophiles. The outcome is a unique platform for developing probes with broad or narrow bioactivity to profile or ligand, respectively, nucleophilic sites on well-validated as well as poorly annotated protein targets. We highlight a suite of biological applications for SuTEx chemistry and discuss future goals for this enabling covalent chemistry.

## General features of activity-based probes

Activity-based probes (ABP) serve as a principal means for selective labeling of functional enzymes and other proteins in complex biological systems. Target labeling by ABPs is facilitated by (i) active- and binding-site accessibility and (ii) reactivity of catalytic or general nucleophilic residues, respectively. ABPs are designed with appropriate electrophiles and photoreactive groups (for proteins devoid of a reactive residue for targeting by ABPs), molecular recognition elements, and reporter tags for detecting activity and function of proteins that are dynamically regulated in cells. A comprehensive review on ABPs and their applications is beyond the scope of this review, however, these are described in detail in other review articles.^5,17^

ABPs share a common design that can be categorized by three components: (1) a reactive group, (2) a binding or affinity element, and (3) a reporter tag for detection and enrichment/identification of probe-modified proteins (Fig. 1). The reactive group (also referred to as the warhead) facilitates covalent modification of the probe to a reactive amino acid residue on the target protein. The recognition (binding/affinity) element, which also serves as a linker for separating the reactive- and reporter-group, is designed to direct ABPs to targets of interest for increased specificity, which is important given the large number of nucleophiles in the human proteome (*e.g.* ATP binding element for kinase-directed ABPs^76^). A basic linker, such as an alkyl or polyethylene glycol (PEG) chain, can also be implemented to develop general ABPs that broadly target enzyme and other protein classes. Examples of enzyme classes for which ABPs have been reported include serine hydrolases,^19,77,78^ kinases,^30,76,79,80^ phosphatases,^81^ cysteine proteases,^82^ metallohydrolases,^83,84^ and E3 ligases.^85,86^ ABPs facilitate broad coverage across a protein class to enable discovery of functional and dysfunctional enzyme activities in disease settings, and for evaluating potency and selectivity of inhibitors against the most relevant targets (*i.e.* mechanistically related proteins or enzymes) directly *in vitro* (protein lysates and cells) and *in vivo* (animals). Tailored ABPs can be developed to react with a limited number of enzymes to detect activities of low abundance enzymes that are masked by higher abundance enzymes for profiling and immunofluorescence studies.^87,88^

The reporter tag facilitates the visualization or purification of probe-labeled proteins (Fig. 2). Fluorophore-conjugated ABPs provide a convenient means for rapid gel-based assays of a moderate number of enzymes (>20) using sodium dodecyl sulfate polyacrylamide gel electrophoresis (SDS-PAGE) and in-gel fluorescence scanning. Biotinylated- or desthiobiotinylated-ABPs (to allow enrichment and elution of ABP-modified molecules) can be used with avidin-affinity chromatography and liquid chromatography-mass spectrometry (LC-MS) for enrichment, identification, and quantification of probe-modified proteins from complex samples. LC-MS ABP methods can be configured to identify and quantify probe- and -inhibitor (by displacement of ABP probe labeling) site of binding by using trypsin digestion prior to avidin affinity chromatography to enrich probe-modified peptides from target proteins (Fig. 2B). Due to the large size and poor cell permeability of fluorophores and biotin, reporter-tagged ABPs generally show poor cellular uptake and tissue distribution and are used predominantly for chemical proteomic studies *in vitro*.

To enable ABP studies in cells and animals, alkynes have been employed as latent reporter tags because of their minimal effects on the physicochemical properties of ABPs containing this functional group. Through the use of copper(i)-catalyzed azide–alkyne [3+2] cycloaddition (CuAAC,^89,90^Fig. 1), an azido-derivatized reporter tag can be conjugated to an alkyne-modified ABP *ex vivo* to allow an efficient and modular biorthogonal reaction for detecting ABP-modified proteins from treated cells and animals.^91–93^ Conversion of inhibitors into ‘clickable’ probes through the incorporation of an alkyne tag also provides a means for evaluating selectivity in proteomes (through direct modification in contrast with competition of ABP labeling) that is complementary to competitive ABPP with class-specific ABPs.^88,94–96^

## Development of SuTEx chemistry

### Triazoles as a tunable leaving group

Initial studies on triazole ureas demonstrated that the triazole heterocycle can serve as a tunable leaving group (LG) for developing serine hydrolase (SH) inhibitors.^20^ The SH family is a large and diverse enzyme family comprised of ∼240 human enzymes (∼1% of the human proteome), which can be subdivided into serine proteases and metabolic serine hydrolases.^18,61,97^ SH enzymes catalyze diverse biochemical functions in physiological and pathological processes through their role as lipases, esterases, amidases, peptidases, and proteases. SHs generally adopt an α,β-hydrolase fold and employ a serine–histidine–aspartate catalytic triad resulting in an activated serine nucleophile for hydrolysis of esters, thioesters, and amide bonds in both protein and small molecule substrates. The enhanced nucleophilicity of the base-activated catalytic serine makes it highly amenable to covalent modification by electrophilic ABPs and inhibitors.^61^

Development of affinity labeling agents^98^ led to the discovery of 1,2,3-triazole ureas (1,2,3-TUs) as potent and selective irreversible inhibitors that inactivate SHs through carbamoylation of the catalytic serine^20,88,95,99–104^ (Fig. 1). Importantly, an evaluation of different heterocyclic LGs showed that triazoles exhibited the appropriate degree of electrophilicity to modify SHs in proteomes while minimizing cross-reactivity outside of the SH protein class.^20^ Both 1,2,3- and 1,2,4-triazoles^105–107^ can serve as an effective LG for SH inhibitor development, and recent evidence supports that regioisomers can affect the activity of resulting compounds.^108^ Combined with the synthetic accessibility of 1,2,3- and 1,2,4-triazoles^109,110^ using diverse methodologies,^111–116^ the triazole moiety offers multiple opportunities for tuning reactivity of electrophilic compounds including the type and position of functional group modifications and selection of regioisomers.

### SuTEx ABP design

**Fig. 3:**
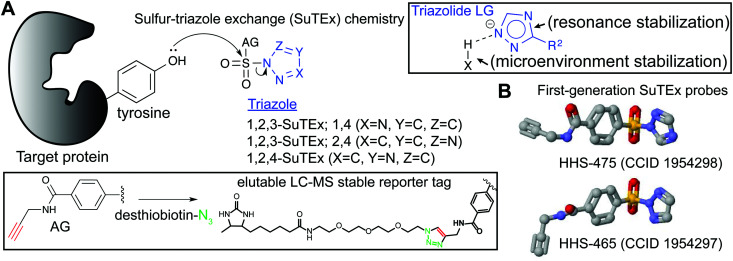
Sulfur–triazole exchange (SuTEx) chemistry. Sulfonyl-triazoles represent a new class of electrophiles that facilitate covalent reaction with tyrosines and lysines through a nucleophilic substitution reaction that is facilitated by a triazolide leaving group (LG). Both intrinsic (resonance stabilization) and extrinsic factors (protein microenvironment) are proposed to contribute to the LG ability of the triazole. Triazole regioisomers (1,2,3- and 1,2,4-isomers) can serve as the LG for development of SuTEx ABPs. The AG of SuTEx ABPs contains an alkyne group for CuAAC conjugation with desthiobiotin-azide to produce a LC-MS stable reporter tag. The selection of a desthiobiotin affinity handle allows enrichment and release of ABP-modified peptides from avidin-agarose beads during preparation of proteomes for LC-MS studies. (B) Crystal structures of first-generation SuTEx ABPs HHS-475 and HHS-465, which differ by the triazole regioisomer selected as the LG.

SuTEx probes are composed of an adduct- (AG) and leaving-group (LG), which denote the compound regions that remain covalently bound and depart, respectively, after reaction with a nucleophilic residue on a protein site (Fig. 3A). Initial SuTEx probes were designed with features to enable global and quantitative – when combined with protein^117,118^ or peptide^119,120^ isotopic labeling strategies – evaluation of the activity of the sulfur electrophile in the proteome. Specifically, first-generation SuTEx ABPs were designed without elaborate binding elements in an effort to assess reactivity mediated by the triazolide LG (Fig. 3A). The minimalistic design of SuTEx ABPs helps facilitate site of binding identifications because larger covalent probes can produce high molecular weight probe-peptide adducts that require manual evaluation of non-traditional fragment ions by LC-MS.^121,122^ Thus, the criteria for selection of the AG included molecular features (size and hydrophobicity) amenable for reverse phase LC and sufficient stability for LC-MS detection and tandem MS/MS identification (Bottom inset, Fig. 3A).

With these goals in mind, first generation SuTEx probes were developed using 4-(chlorosulfonyl)benzoic acid as a common precursor for stepwise addition of propargylamine followed by coupling to an unsubstituted 1,2,3- or 1,2,4-triazole(HHS-465 and HHS-475, respectively; Fig. 3B). Akin to global covalent probes of cysteine (iodoacetamide, IAA^22,23,25^) and lysine (sulfotetrafluorophenyl ester, STP^28^) nucleophiles, we observed broad reactivity of HHS-465 and HHS-475 in cell proteomes and identified tyrosines and lysines as the principal sites of binding in domains involved in enzymatic, nucleotide recognition, and protein–protein interaction function.^36^ Importantly, SuTEx probes can be used to profile accessibility and reactivity of thousands of binding sites in live cells, which should prove useful for investigating regulation of protein function that can only be captured in a cellular context.^36^

The ability to accurately assign tyrosine and lysine modification sites on proteins in the complex milieu of many nucleophilic residues found in cellular proteomes was enabled by the predictable MS/MS probe-modified peptide fragmentation and LC-MS compatible reporter-tagged AG for chemical proteomic evaluation of SuTEx probe-peptide adducts^36^ (Fig. 3A). These initial chemical proteomic studies also provided clues to the influence of the LG on the selectivity of the SuTEx probe activity against tyrosine compared with lysine residues. Specifically, the 1,2,4-SuTEx probe HHS-475 showed increased preference for tyrosine over lysine modification when compared with its 1,2,3-SuTEx counterpart (HHS-465, Fig. 3B).

### Tuning the SuTEx scaffold for tyrosine chemoselectivity

**Fig. 4:**
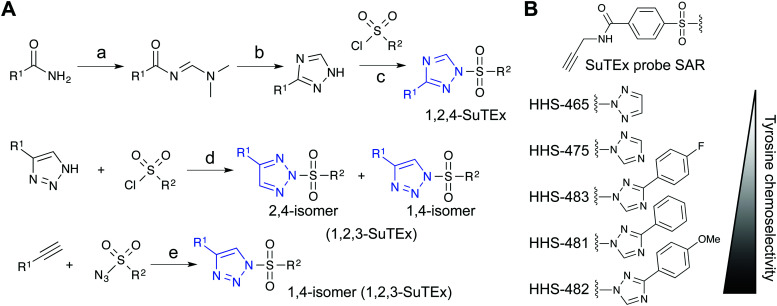
Synthetic routes for generating triazole diversity for developing SuTEx probes. (A) Examples of synthetic routes to generate 1,2,3- and 1,2,4-triazole chemical diversity for developing SuTEx ABPs and ligands. The following experimental conditions are used for the synthetic reactions shown: (a) DMF–DMA, 120 °C, 3 hours; (b) Hydrazine, acetic acid, 90 °C, 1.5 hours; (c) ethanol; (d) THF/Et_3_N room temperature, overnight, 1.1 equivalents triazole; (e) toluene, copper(i) thiophene-2-carboxylate (CuTC^175^), 12–48 hours, 1 : 1 ratio of alkyne and azide. (B) SuTEx ABPs with modifications on the triazolide LG. SuTEx ABPs containing the 1,2,4-triazolide LG show improved specificity for tyrosine compared with lysine modifications on protein sites and this chemoselectivity can be further augmented with functional group modifications as shown.

Initial evaluation of LG modifications on proteome activity of SuTEx ABPs focused on expanding the 1,2,4-triazole series because of its augmented tyrosine selectivity. The modular nature of assembling SuTEx molecules takes advantage of established synthetic methods for accessing unsubstituted and modified triazole regioisomers^109–116^ to greatly expand the chemical diversity of resulting ABPs^36^ and ligands^123^ (Fig. 4A). A series of SuTEx probes bearing phenyl-substituted 1,2,4-triazoles containing electron withdrawing (EWG) and electron donating (EDG) groups were synthesized and tested by quantitative LC-MS chemical proteomics. Proteome-wide structure–activity relationship (SAR) studies were expedited by using a common AG to test changes in reactivity and specificity due to exclusive modification on the LG (Fig. 4B).

Our SAR studies revealed that incorporation of a triazolide LG significantly increased reactivity (∼4-fold) of the SuTEx electrophile compared with that of the SuFEx counterpart.^36^ Modifications to the triazolide LG affected preference for tyrosine compared with lysine modification (Y/K ratio) as well as overall reactivity as determined by the number of modified Y and K sites.^36^ For example, the addition of a phenyl group increased both the Y/K ratio and total number of modified sites. Comparison of EWG (*para*-fluoro) and EDG (*para*-methoxy) groups showed that the former increased overall reactivity but lowered tyrosine specificity of SuTEx probes.^36^ From these studies, we identified HHS-482 as a tyrosine chemoselective probe that exhibited high tyrosine specificity (∼75% of ABP-modified sites) while retaining overall reactivity that was comparable with HHS-475^36^ (∼3000 total probe-modified sites, Fig. 4B).

To understand the tyrosine chemoselectivity of SuTEx probes, we performed reactivity studies in solution using high-performance liquid chromatography (HPLC) to test HHS-475, HHS-482, and the SuFEx counterpart, HHS-SF-1, against nucleophiles that mimic tyrosine (*p*-cresol) and lysine (*n*-butylamine) side chains. The HPLC solution studies provided further insights into common and distinguishing features of SuTEx compared with SuFEx ABPs. First, SuTEx probes are stable in solvent vehicles (*e.g.* dimethyl sulfoxide or DMSO) typically used to deliver compounds in biological experiments. Second, the triazolide LG augmented reactivity of sulfonyl probes for the phenol nucleophile. Finally, the differences in tyrosine chemoselectivity between SuTEx probes (*e.g.* between HHS-482 and HHS-475) cannot be explained by solution reactivity and is likely mediated by the protein site environment.^36^

### Comparison of SuTEx with other sulfur electrophiles

A principal differentiator of SuTEx and SuFEx chemistry is the choice of LG for facilitating nucleophilic substitution reactions at the SO_2_ center. Akin to SuFEx, both direct substitution and addition-elimination pathways are potential mechanisms for explaining the substitution reaction of sulfonyl-triazoles.^73,124,125^ Elucidating the mechanistic details of SuTEx reaction will likely provide insights into how this electrophile mediates specificity for reaction with the phenol nucleophile of tyrosines. Although details of the reaction mechanisms are not completely understood, we highlight several features of SuTEx chemistry that merit its utility for biological applications. Solution and proteome reactivity studies demonstrate that the triazole heterocycle serves as an effective LG for driving reactivity of SuTEx chemistry with – to the best of our knowledge – exclusive reaction at the sulfur center.^36,123^ Notably, the increased reactivity of SuTEx in the presence of nucleophiles both in solution and proteomes did not compromise the overall stability in organic and aqueous solvents, which is an important feature for developing ABPs^36^ and ligands^123^ for biological studies.

The increased reactivity of sulfur electrophiles using a triazolide compared with a fluoride LG can be potentially explained by the distinct attributes that result from deploying a heterocyclic LG. Stabilization of the developing fluoride ion, *e.g.* by protic centers in protein sites, in the substitution process is key for driving the SuFEx reaction (described in detail in the following review^73^). In addition to hydrogen bonding, the departing triazolide in a SuTEx reaction can be further stabilized by resonance (Top inset, Fig. 3A). The departing triazolide would presumably resemble the anion from deprotonation of the pyrrole-type nitrogen of triazoles, which has been shown to be stabilized by aromaticity.^126^ The unusual stability of a triazole – resulting from its aromatic character – also makes it resistant to hydrolysis and oxidative/reductive conditions,^127–129^ which can be liabilities when developing chemical probes for studies in living systems. The ability of the triazole to facilitate hydrogen bonding and dipole–dipole interactions are also advantageous for mediating binding recognition at protein sites.

In summary, the triazole heterocycle is well-suited as a tunable LG for driving nucleophilic substitution at the SO_2_ center in reactions with protein sites because of its intrinsic stability and propensity for participating in intermolecular interactions with the local microenvironment. Of interest for future studies, the aromaticity of azoles is affected by electronegativity differences between adjacent atoms^130^ and suggests further tunability by extending the principles of SuTEx to other heterocycle LGs.

## Biological applications of SuTEx chemistry

### Identifying hyper-reactive tyrosines for functional and pharmacological investigations

The local microenvironment of proteins can alter reactivity of amino acid side-chains resulting in specialized sites that are enriched for functional residues.^131^ Reactivity of these nucleophilic sites can vary by several orders of magnitude for a given residue depending on the local microenvironment of a protein. A prominent example is the catalytic serine of SHs that displays heightened nucleophilicity that is facilitated by a catalytic triad (*e.g.* serine-histidine-aspartate^132,133^). This remarkable activation of the serine nucleophile enables this enzyme class to cleave a variety of substrates that contain amide, ester, and thioester bonds.^61^ These sites with heightened nucleophilicity – or ‘hyper-reactivity’ – have been reported for additional residues including cysteine,^22^ lysine,^28^ and methionine.^35^ Hyper-reactive sites typically represent only a small fraction of amino acid residues and are localized in functional protein domains involved in catalysis, protein–protein interactions, and ligand binding.

**Fig. 5:**
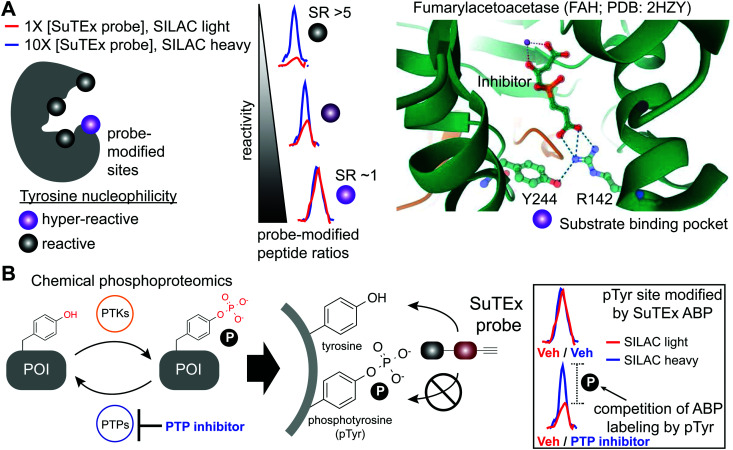
Application of SuTEx for functional profiling of hyper-reactive and phosphorylated tyrosines. (A) A subset of tyrosine sites on proteins exhibit augmented reactivity due to the local microenvironment. For example, nearby basic residues (R142) can mediate electrostatic stabilization of the phenolate anion (Y244) and effectively increase the nucleophilic character of this side chain under physiological conditions (hyper-reactive Y244 site of FAH shown as an example; PDB: 2HZY). Quantitative chemical proteomics using SuTEx ABPs can identify these hyper-reactive tyrosine sites on a global scale by comparing probe labeling at low and high ABP concentrations. Hyper-reactive tyrosines are expected to saturate probe labeling at low (light) and high (heavy) concentrations resulting in comparable enrichment of isotopically labeled (*e.g.* SILAC) ABP-modified peptides. SILAC peptide ratios (SR; calculated from area under the curve of light and heavy peptide abundances) can be used to rank tyrosine sites based on reactive (SR > 5) and hyper-reactive (SR ∼1) character. (B) Tyrosine phosphorylation on proteins is a post-translational modification that is dynamically regulated by protein tyrosine-kinases (PTKs) and -phosphatases (PTPs). Cellular treatment with global PTP inhibitors such as pervanadate are expected to shift the equilibrium towards accumulation of phosphotyrosine (pTyr) modifications, which would effectively block SuTEx ABP labeling. This competitive assay format represents the basis of a SuTEx chemical phosphoproteomics strategy to profile pTyr regulation in live cells. Veh: vehicle.

Although catalytic serines of SHs can be identified from canonical sequence motifs, the primary sequence surrounding non-conserved hyper-reactive residues, *e.g.* cysteines and lysines, are mediated by structural features that are difficult to predict from the protein sequence alone. The intrinsic reactivity of amino acid residues in proteomes can be compared *en bloc* by measuring concentration-dependent labeling with reactive covalent probes.^22,134,135^ Amino acid residues that are hyper-reactive are expected to be probe modified to a similar extent at low and high probe concentrations due to saturation, and less nucleophilic sites should exhibit concentration-dependent changes in probe labeling (Fig. 5A). Tyrosine-reactive SuTEx ABPs combined with stable isotope labeling with amino acids in cell culture (SILAC) quantitative proteomics was used to globally assess whether tyrosines differ in intrinsic nucleophilicity and to determine the diversity of protein domains that contain these hyper-reactive sites.^36^ Across thousands of SuTEx ABP-modified sites, a small subset of hyper-reactive tyrosines that showed enhanced nucleophilicity (∼5% of all quantified tyrosine sites) were identified. The majority of proteins possessed a single hyper-reactive tyrosine among several SuTEx ABP-modified tyrosine sites quantified. As expected, hyper-reactive tyrosines were enriched for enzyme domains but were also found in protein–protein interaction and ligand-binding domains on proteins.^36^

Tyrosine reactivity annotations were verified in biochemical assays to identify, for example, a catalytic tyrosine in the glutathione binding site (G-site) of glutathione *S*-transferase Pi (GSTP1).^136,137^ A hyper-reactive tyrosine site (Y475) was discovered in the poorly characterized Yjef-N domain of the scaffolding protein enhancer of mRNA decapping protein 3 (EDC3).^138–140^ The findings from the above two examples highlight the versatility of SuTEx for identifying reactive tyrosine sites on enzymes as well as proteins involved in assembly of cytoplasmic RNA–protein (RNP) granules, known as P-bodies, in post-transcriptional regulation.^141–143^ Data from studies using SuTEx and SuFEx probes have revealed features of tyrosine sites that exhibit enhanced nucleophilicity. For example, our previous studies identified several arginines (R548 and R572) in proximity to Y417 of dipeptidyl peptidase 3 (DPP3), which exhibits moderate nucleophilicity in our chemical proteomic studies.^36^ A dramatic example of potential base activation of the phenol on a tyrosine can be seen from crystal structures showing hydrogen bonding between R142 and Y244 of fumarylacetoacetase that was identified as hyper-reactive by SuTEx chemical proteomics^144^ (FAH, Fig. 5A). Cationic residues have been shown to perturb the p*K*_a_ of neighboring tyrosine residues to activate these sites for catalytic functions.^145,146^

Studies using arylfluorosulfates probes have identified examples of chemoselective modification of protein tyrosines sites; nearby cationic residue (*e.g.* arginine or lysine) facilitate covalent reaction by lowering the p*K*_a_ of the tyrosine phenol to catalyze the SuFEx reaction.^37,147^ Specifically, the pH dependence for SuFEx probe modification was used to identify a p*K*_a_ perturbed Y134 site due to nearby arginine residues on cellular retinoic acid binding protein 2 (CRABP2).^37^ The lowered p*K*_a_ of Y134 (p*K*_a_ of ∼7.6) could explain the selective modification of this site on CRABP2 by SuFEx probes due to its enhanced reactivity at near-neutral pH. Mutagenesis of these arginine residues greatly impaired SuFEx probe labeling of CRABP2 Y134 even at elevated pH, which suggests a further role for these cationic residues in catalyzing covalent reaction potentially through stabilization of the fluoride LG. Additional examples of SuFEx-modified tyrosine sites that appear to be dependent on nearby cationic residues for activation were reported for GSTP1 and other protein targets.^147^

As SuTEx ABPs become more widely adopted in the chemical biology community, we envision a deeper understanding of molecular features that describe – and potentially predict – ‘hyper-reactive’ tyrosine sites in the human proteome. An interesting feature that emerged from these initial studies was the observation of an inverse relationship between tyrosine reactivity and evidence of phosphorylation. Specifically, tyrosines with low reactivity were significantly overrepresented as reported phosphotyrosine (pTyr) sites compared with more reactive tyrosine sites.^36^ A potential explanation of lowered reactivity of pTyr sites is to minimize non-specific covalent modification that could negatively impact cell biology given the critical role of pTyr signaling in cells.^148^

In summary, the proteome is abundant with reactive tyrosines. A small subset of these nucleophilic residues exhibit features of hyper-reactivity that endows proteins with capabilities for catalysis and protein–protein and protein-small molecule recognition, and therefore are potential sites for pharmacological modulation.

### Chemical phosphoproteomics for investigating phosphotyrosine regulation

The broad tyrosine coverage of SuTEx was recently adapted for development of an antibody-independent chemical phosphoproteomic method. Strategies capable of overcoming the low abundance and substoichiometric phosphorylation-site occupancy of phosphoproteins^149–151^ are needed to enable functional phosphoproteomic profiling. This is especially true for tyrosine phosphorylation. Compared with phospho-serine and -threonine, pTyr modifications represent a rare subset (∼1%) of the human phosphoproteome.^152,153^ The lower frequency of pTyr modifications is due to several features of this phosphoresidue including: (1) pTyr is more prominent on sites for protein regulation and not structural function, (2) pTyr is tightly regulated and activated only under specific conditions, and (3) pTyr sites have a very short half-life.^148,154^ Various enrichment strategies have been pursued for overcoming the sensitivity issues with pTyr analyses.^149,151^ The most widely-adopted method utilizes a common set of anti-pTyr antibodies for phospho-peptide or -protein enrichment in conjunction with mass spectrometry for phosphoproteomics.^155–157^ SuTEx offers a complementary methodology that adopts a competitive format to identify putative pTyr sites that does not require antibodies.

SuTEx phosphoproteomics is based on the premise that the ability of an ABP to modify a tyrosine site is competed by activation of phosphorylation (Fig. 5B). As proof of concept, cell treatments with a global protein tyrosine phosphatase inhibitor pervanadate, which results in activation and accumulation of pTyr modifications across the proteome was performed to identify pervanadate-sensitive (PerS) sites that represented putative pTyr sites. Across thousands of quantified sites, a small subset of PerS sites (∼3% of all quantified tyrosines) was identified, which is in agreement with the low frequency of tyrosine phosphorylation compared with the more abundant phospho-Ser and -Thr residues.^152,153^ In support of SuTEx as a chemical phosphoproteomics technology, the probe-modified tyrosine sites identified in the PerS group were enriched for annotated pTyr modifications when cross-referenced against the PhosphoSitePlus^158^ (HTP > 10) database, and represented only a small fraction of all unique SuTEx ABP-modified tyrosines detected.^36^ The abundances of the majority of probe-modified tyrosine sites were unchanged between vehicle- and pervanadate-treated cells, which supports the capability of the SuTEx platform to capture rare post-translational events such as pTyr modifications.

PerS sites were confirmed as authentic pTyr sites for several proteins by demonstrating that the observed blockade of SuTEx ABP labeling was a result of direct phosphorylation at respective tyrosine sites. For example, competition of SuTEx ABP labeling at Y705 and Y228 of STAT3 and CTNND1, respectively, was directly anticorrelated with phosphorylation at these same sites as determined by western blots with anti-pTyr antibodies. Both STAT3 and CTNND1 sites are highly annotated pTyr sites and reported substrates for tyrosine kinases in cancer cell signaling^159,160^ and our results are in agreement with the cell type used for these phosphoproteomic studies. By comparison, the pervanadate-insensitive Y105 site of PKM did not show changes in phosphorylation or ABP labeling with pervanadate treatments, which further supports SuTEx probe labeling as a means to evaluate the phosphorylation state of tyrosine residues.^36^

Collectively, these proof of concept studies demonstrate that SuTEx chemical proteomics is a complementary strategy for quantifying pTyr status through competitive probe labeling at tyrosine sites of interest. Considering that tyrosines are subject to several post-translational modifications (*e.g.* glycosylation,^161^ sulfation^162^ and nitration^163^), SuTEx chemical proteomics may have broader applications for probing tyrosine regulation in cell biology.^164^

### Fragment-based ligand discovery

**Fig. 6:**
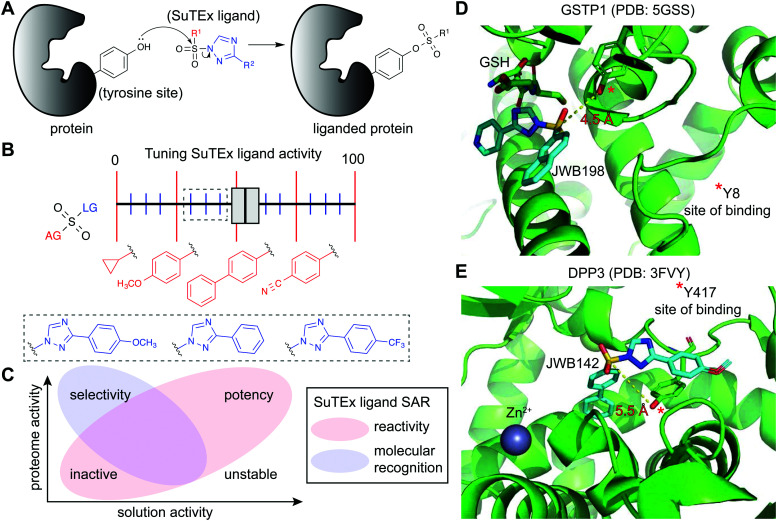
Enabling fragment-based ligand discovery (FBLD) for developing protein inhibitors using SuTEx. (A) SuTEx compounds can be used to inactivate proteins by covalent modification of tyrosines in binding sites mediating catalytic and non-catalytic functions. (B) The reactivity of the sulfur electrophile can be tuned by functional group (FG) modifications on the adduct- (AG) and leaving-group (LG) of SuTEx ligands. The magnitude of effects can be adjusted based on modifications to the AG (red FGs) and LG (blue FGs) for coarse and fine ‘tuning’ of SuTEx ligand activity. (C) Comparison of solution and proteome activity of SuTEx ligands provide insights into structure–activity relationships (SAR) to guide development of selective protein inhibitors. (D) Docked structure of GSTP1 (PDB: 5GSS) with JWB198 in the presence of glutathione (GSH) substrate. Distance from the phenol nucleophile of Y8 to the sulfur electrophile of JWB198 are depicted with a yellow dashed line (4.5 Å). Docking was performed with glutathione removed from the protein structure. (E) Docked structure of DPP3 (PDB: 3FVY) with JWB142. Distance from the phenol nucleophile of Y417 to the sulfur electrophile of JWB142 are shown with a yellow dashed line (5.5 Å). All docking was performed using AutoDock Vina with the binding region set as a distance of 25 Å from the ABP-modified tyrosine. The docked structures shown represent the conformation with the minimum distance between the electrophile and the liganded tyrosine site prior to covalent reaction.

SuTEx chemistry is well-suited for developing protein ligands when combined with ABPs in a competitive format (Fig. 2). Using this approach, candidate ligands can be screened against multiple proteins simultaneously in cell lysates and live cells and can facilitate evaluation of potency and selectivity of putative inhibitors in a single experiment.^5^ The diversity of synthetic routes to modify substituents on the AG and LG using different triazole regioisomers greatly expands medicinal chemistry opportunities for tuning reactivity and molecular recognition of SuTEx ligands (Fig. 6A). Importantly, the use of a common scaffold for developing ABPs (alkyne modified) and protein ligands (functional group modified) helps ensure that the most relevant protein targets (and off-targets) are not missed in competitive profiling experiments. As described above, hyper-reactive tyrosines are localized to functional domains and serve as promising sites for developing protein ligands because of their propensity to react with electrophiles. As a result, these nucleophilic tyrosines can facilitate prioritization of targets for medicinal chemistry efforts to develop covalent ligands with properly balanced reactivity and proteome-wide specificity for modulating protein function.

Initial efforts to explore SuTEx chemistry for fragment-based ligand discovery (FBLD^165–167^) focused on evaluating the sensitivity of the sulfur electrophile to functional group modifications on the AG and LG.^123^ Considering the broad reactivity of SuTEx ABPs, SAR studies were implemented to determine whether potency and specificity against a protein tyrosine site can be augmented through modifications to the AG, LG, or both regions. A library of fragment electrophiles was developed using the tyrosine chemoselective probe HHS-482 as the base scaffold for medicinal chemistry.^123^ Competitive profiling of this initial SuTEx fragment library identified over 300 liganded tyrosine sites across hundreds of distinct protein targets quantified by chemical proteomics. Initial SAR revealed that the EWG and EDG character of functional groups can affect reactivity and selectivity of SuTEx fragment ligands against tyrosine and lysine sites on proteins. The magnitude of effects was dependent on the region of modification; the SuTEx electrophile was generally more sensitive to AG compared with LG modifications.^123^ An outcome from these findings was the proposed concept of “coarse” and “fine” tuning of SuTEx ligand activity through AG and LG modifications, respectively (Fig. 6B).

An important finding from these FBLD studies was the stark contrast between reactivity of SuTEx-ABPs compared with -fragment ligands in proteomes. Considering that the fragment ligand library was based on the HHS-482 structure (parent ABP), we expected to identify fragment ligands capable of effectively competing with labeling of the parent ABP in our LC-MS studies. Surprisingly, the most reactive fragment ligand identified (JWB150) was only capable of competing at <20% of all HHS-482-modified sites quantified by chemical proteomics. A potential explanation is that the reporter tag itself, which is coupled through an electron withdrawing carbonyl group (Fig. 3), is potentiating reactivity of SuTEx ABPs. Our recent studies exploring various linkers for appending the alkyne group in SuTEx ABPs support this hypothesis.^168^ Another key finding was the near-complete loss of compound reactivity when the AGs of SuTEx ligands were modified with alkyl substituents, which further illustrates the tunable nature of SuTEx for developing protein ligands.^123^

By varying aryl substituents on the AG and LG, SuTEx ligands could be tuned for inhibitory activity (>75% reduction in enrichment by HHS-482 ABP) against a small subset of tyrosine sites (∼7–13 probe-modified sites^123^). These findings were important for establishing feasibility of using SuTEx chemistry to develop covalent ligands directed at specific proteins and binding sites. Liganded tyrosine sites were enriched for functional domains involved in nucleotide binding, protein–protein interactions, metal binding, and enzymatic reactions, and a large fraction of these target proteins lacked known pharmacological probes.^123^ Notably, comparison of enriched domains from SuTEx ABP- *versus* ligand-modified tyrosine sites revealed distinct binding profiles. These findings support molecular recognition as an important contributor of protein–ligand interactions of SuTEx compounds in complex proteomes (Fig. 6C). Further support for binding recognition was apparent from examples of SuTEx ligands that exhibited higher reactivity in solution but substantially reduced activity against protein sites compared with structurally analogous compounds (*e.g.* JWB152 *vs.* JWB150^123^).

In summary, SuTEx chemistry is well-suited for developing ABPs and matching ligand compounds for FBLD to study functional protein sites. The ability to seamlessly transition between SuTEx ABPs and protein ligands provide a unified platform to scan for tyrosines (and lysines) that are ligandable^169^ (and potentially druggable) in cell lysates and live cells.

### Developing protein inhibitors by liganding catalytic and non-catalytic tyrosines

The FBLD studies above identified opportunities for testing SuTEx ligands as inhibitors that block protein function by modifying tyrosines in binding sites. A competitive gel-based screen (Fig. 2) using the HHS-482 ABP was implemented to identify ligands capable of modifying the single hyper-reactive tyrosine site of GSTP1 (Y8), which is catalytic and a reported phosphorylation site.^36,136,137^ From these screening efforts, JWB152 and JWB198 emerged as lead inhibitors against recombinant GSTP1.^123^ Given that co-crystal structures of GSTP1 and SuTEx ligands are not currently available, we performed molecular docking^170^ to predict how JWB198 binds in the G-site (containing the ABP-modified Y8) of GSTP1. We identified a ligand binding conformation that places the phenol nucleophile of the catalytic Y8 in proximity to the electrophilic sulfur (∼4.5 angstroms). Of interest for future inhibitor design, the JWB198 binding conformation would result in direct competition with the glutathione substrate of GSTP1 (Protein Data Bank (PDB) accession code: 5GSS;^171^Fig. 6D).

Although JWB152 and JWB198 displayed comparable inhibitory activity *in vitro* by chemical proteomics and biochemical assays, only JWB198 could ligand the Y8 site of GSTP1 in live cells.^123^ Further exploration of proteome-wide specificity revealed that JWB198 showed >3-fold reductions in general cross-reactivity against protein tyrosine sites (compared with JWB152) while still maintaining ∼70% blockade of GSTP1 Y8 in cells. Inhibition was site-specific as determined by lack of activity of JWB198 against other GSTP1 ABP modified sites (Y50, Y64, Y80, Y119, and Y199). These findings highlight the importance – and enabling capability of SuTEx chemistry – for tuning the reactivity of sulfur electrophiles to reduce general cross-reactivity in living systems. A benefit of improved specificity is increasing the intracellular fraction of compound that can effectively engage a target protein site by reducing occupancy at additional cellular proteins.^172^

SuTEx can be effective for developing ligands against non-catalytic sites containing tyrosines that may not be inherently hyper-reactive. As a proof of concept approach, a screening method was used to identify ligands for Y417, which is located near the zinc-binding region of dipeptidyl peptidase 3 (DPP3). Interestingly and for reasons that require further investigation, DPP3 is among a collection of proteins that are modified at a single tyrosine site by SuTEx ABPs. The Y417 site of DPP3 is not catalytic because mutation of this residue to a phenylalanine results in a mutant protein with biochemical activity comparable to wild-type protein.^36^ Although mutagenesis did not impact activity, covalent modification of Y417 with a ligand was predicted to occlude access to the active site. This strategy is analogous to the success of covalent ligands developed to modify non-catalytic cysteine residues of kinases^173^ and other protein families.^174^ The identification of JWB142 as a first-in-class covalent DPP3 inhibitor through blockade of Y417 supports liganding non-catalytic tyrosines using SuTEx as a strategy for developing protein inhibitors.^123^ As an initial step to understand the binding mode, we performed molecular docking of JWB142 in the zinc binding pocket of DPP3 to predict a ligand conformation that places the phenol nucleophile of the non-catalytic Y417 in proximity to the electrophilic sulfur (∼5.5 angstroms, PDB accession code: 3FVY; Fig. 6E).

## Conclusions and future outlook

In summary, the SuTEx electrophile has emerged as a versatile chemical biology tool that is well-suited for global investigations of tyrosines in protein functional sites. In this review, we highlighted unique features of sulfonyl-triazoles that enable ABP and ligand development for chemical proteomic and biological applications. The ability to modify the AG and LG with different outcomes in reactivity and specificity enhances the tunable nature of SuTEx for tailoring the activity of probes for the intended biological target and system. The findings to date have provided a glimpse of the potential for using this electrophile for chemical proteomic evaluation and pharmacological modulation of reactive tyrosines on proteins with functions ranging from enzyme chemistry to protein–protein and protein–nucleotide recognition.

The ability to differentiate tyrosines based on nucleophilicity in a quantitative fashion across the proteome allows prioritization of target proteins and sites based on function and ligandability. Hyper-reactive tyrosines, akin to the well-studied catalytic serine of SHs, are prime candidates for developing pharmacological agents to perturb catalytic and non-catalytic protein functions.^36^ Conversion of SuTEx ABPs to protein ligands requires only a facile removal of the alkyne reporter group, which consequently opens another site for incorporating functional group diversity. A key discovery was the stark difference in reactivity and binding specificity of SuTEx ligands compared with ABPs despite using a common scaffold for medicinal chemistry efforts.^123^ Thus, the combination of SuTEx ABPs and ligands – and capability to seamlessly switch between these probe formats – can be used to scan for hyper-reactive tyrosine sites (and proteins with a single modified tyrosine) that can be liganded for developing cell-active protein function modulators.

Competition of SuTEx ABP labeling can be exploited to quantify whether a tyrosine site of interest is phosphorylated upon blockade of PTPs. The prospect of developing SuTEx into a chemical phosphoproteomics technology may uncover novel pTyr sites that escape more traditional antibody-based phosphoproteomic methods. We propose that both approaches would be complementary and highly synergistic given that detection of putative pTyr sites by SuTEx would require verification with standard phosphoproteomics as well as development of antibodies for follow-up biological investigations. The study of annotated pTyr sites found in public repositories such as PhosphoSitePlus could benefit from a chemical approach using SuTEx ABPs to understand specificity of pTyr regulation in live cells when combined with PTP inhibitors. Importantly, the inclusion of SuTEx into standard phosphoproteomic workflows also provides an opportunity to develop ligands that target reported and novel pTyr sites of interest. For example, an interesting future study is to investigate the functional impact of liganding the Y8 pTyr site of GSTP1, which has been implicated in downstream signaling of epidermal growth factor receptor (EGFR),^136,137^ using our recently developed cell-active SuTEx ligand.^123^

The triazole has been shown to serve as an effective LG for facilitating SuTEx reaction against nucleophiles and raises the question of whether other heterocycles could perform a similar – and potentially tunable – role for developing the next-generation of sulfur covalent probes. We find it interesting that the aromaticity of azoles is affected by the electronegativity differences between adjacent atoms.^127,130^ Applying these underlying principles may furnish additional opportunities for tuning the sulfur electrophile through careful selection of azoles to facilitate general sulfur-heterocycle exchange chemistry. Incorporating more automated synthetic and screening approaches, *e.g.* using late stage functionalization (LSF),^75^ can further expand the chemical diversity and target scope of SuTEx probes and potentially advance this strategy for animal studies. For LSF applications, the triazole can also serve as a release linker to develop clickable ABPs for mapping protein targets of drugs and other structurally complex compounds.^168^

The success of SuTEx and SuFEx exemplify the growing number of biological and chemical applications that are possible using the sulfur electrophile. The future is promising as synthetic chemists and chemical biologists continue to innovate in covalent chemistry to usher in a ‘Su’-nami of new sulfur electrophiles for basic and translational discoveries.

## Conflicts of interest

The authors declare no competing financial interest.

## Supplementary Material
